# Traumatic proximal tibiofibular fracture and dislocation

**DOI:** 10.1186/s12891-024-07577-w

**Published:** 2024-06-15

**Authors:** Bo Li, Xuan Tian, Han Fei, Guoshen Li, Xinbao Wu

**Affiliations:** 1 Department of Orthopaedics & Traumatology, Beijing Jishuitan Hospital, Capital Medical University, No. 31, Xinjiekou East Street, Xicheng District, Beijing, China; 2 Department of Vascular Surgery, Beijing Jishuitan Hospital, Capital Medical University, Beijing, China

**Keywords:** Traumatic proximal tibiofibular fracture and dislocation (PTFD), Vascular injury, Knee trauma, Amputation, Emergency

## Abstract

**Background:**

Traumatic proximal tibiofibular fracture and dislocation (PTFD) have been rarely studied and are easily missed in clinical practice. PTFD is considered a marker of severely traumatized knees. The purpose of this study was to retrospectively analyze the incidence and impact of PTFD in traumatized knees with vascular injury.

**Methods:**

Patients with knee trauma and vascular injury were included from January 2022 to October 2023. X-rays and CT scans of included patients were retrospectively analyzed to determine the presence of PTFD. Patients were further divided into PTFD group and non-PTFD group for further comparative analysis.

**Results:**

A total of 27 patients (28 limbs) were included. Incidence of PTFD was 39.3% (11/28) in traumatic knee with vascular injury, including 8 anterolateral dislocations and 3 posteromedial dislocations. PTFD group had significantly more limbs with open injuries compared with non-PTFD group (10/11 VS 7/17, *p*<0.05). Amputation rate of PTFD group was as high as 40% (4/10), compared to 23.5% (4/17) in non-PTFD group. However, the difference between two groups was not statistically significant (*p*>0.05).

**Conclusions:**

PTFD was easily overlooked or missed. In traumatized knees with vascular injury, incidence of PTFD was high. The presence of PTFD might indicate severe knee trauma and the possibility of open injury. Although there was no significant difference compared with non-PTFD group, PTFD group had a relatively high amputation rate of 40%.

## Background

The fibular head is normally located posterolateral to proximal tibia and articulates with it. Dislocation of proximal tibiofibular joint may occur during high-energy injuries. Traumatic proximal tibiofibular fracture and dislocation (PTFD) is relatively rare and can easily be overlooked or missed [1–9]. Most existing relevant studies are case reports [1, 5, 10–14]. In recent years, research has begun to focus on traumatic PTFD. It is considered a marker of severely traumatized knees and may be associated with a higher risk of vascular injury and amputation [15, 16]. The incidence of traumatic proximal tibiofibular dislocation among common tibial plateau and tibial shaft fractures has been reported to be only 1–2% [15]. However, its incidence in higher energy trauma and its effect were still unknown.

The purpose of this study was to retrospectively analyze the incidence of PTFD and its influence in traumatized knees with vascular injury, which usually resulted from high energy trauma.

## Materials and methods

Patients who visited the emergency department of our hospital from January 2022 to October 2023 were reviewed. All cases (≥ 14 years of age) with knee trauma and vascular injury, diagnosed by angiography, were included. Patients who initially presented to other hospitals with undiagnosed vascular injuries were excluded because delayed diagnosis might increase the risk of amputation. X-rays and CT scans of included cases were retrospectively analyzed to determine the presence of PTFD. Patients were further divided into PTFD group and non-PTFD group. Multi-department collaboration (orthopedic trauma, vascular surgery, and microsurgery) determined and implemented the treatment plan. The decision to salvage or amputate a limb during emergency surgery was based on the patient’s hemodynamic stability, limb condition, and Mangled extremity severity score (MESS). For patients undergoing limb salvage, tibial and femoral fractures were reduced and externally fixed, and the injured blood vessels were repaired. If PTFD hindered vascular repair, the proximal tibiofibular joint was reduced first, otherwise no treatment was performed. Approval was obtained from the institutional review boards of Beijing Jishuitan Hospital, Capital Medical University (K2024-035-00), and all procedures used adhere to the tenets of the Declaration of Helsinki.

Patient demographics, mechanism of injury, medical records, imaging data, angiographic results, and treatment outcomes were recorded. Statistical analysis was conducted using SPSS software (V.20.0, Chicago, Illinois, USA). Independent sample t test was used for continuous variables, and chi-square test was used for categorical variables. Data were presented as mean ± SD or percentages.

## Results

A total of 27 patients (28 limbs) diagnosed with knee trauma and vascular injuries were included. The main diagnoses in orthopedic trauma included distal femoral fractures, proximal tibial fractures, or knee dislocations. There was no definite diagnosis of PTFD in any of these case records. By retrospective reviewing emergency X-rays and CT scans again, we found that 11 limbs of 11 patients (mean age 46 ± 13.8, 3 females) had PTFD, and 17 limbs of 16 patients (mean age 44.8 ± 17.1, 3 females) did not have PTFD (Table 1). All the 11 PTFDs occurred together with proximal tibial fractures or distal femoral fractures.

**Table 1:** Comparison between PTFFD group and non-PTFFD group

	PTFFD group	Non-PTFFD group	*P*
Patients	11	16	
Female: Male	3:8	3:13	0.662
Age	46 ± 13.8	44.8 ± 17.1	0.842
Patients with combined head, chest, or abdominal injuries	3/11 (27.3%)	1/16 (6.3%)	0.273
Number of limbs	11	17	
Proportion of open injuries	10/11 (90.9%)	7/17 (41.2%)	0.016*
Popliteal artery injury VS partial branch injury below popliteal artery	8 VS 3	15 VS 2	0.353
Death rate	1/11 (9.1%)	0/16 (0%)	0.407
Amputation rate	4/10 (40%)	4/17 (23.5%)	0.415

**Fig. 1:**
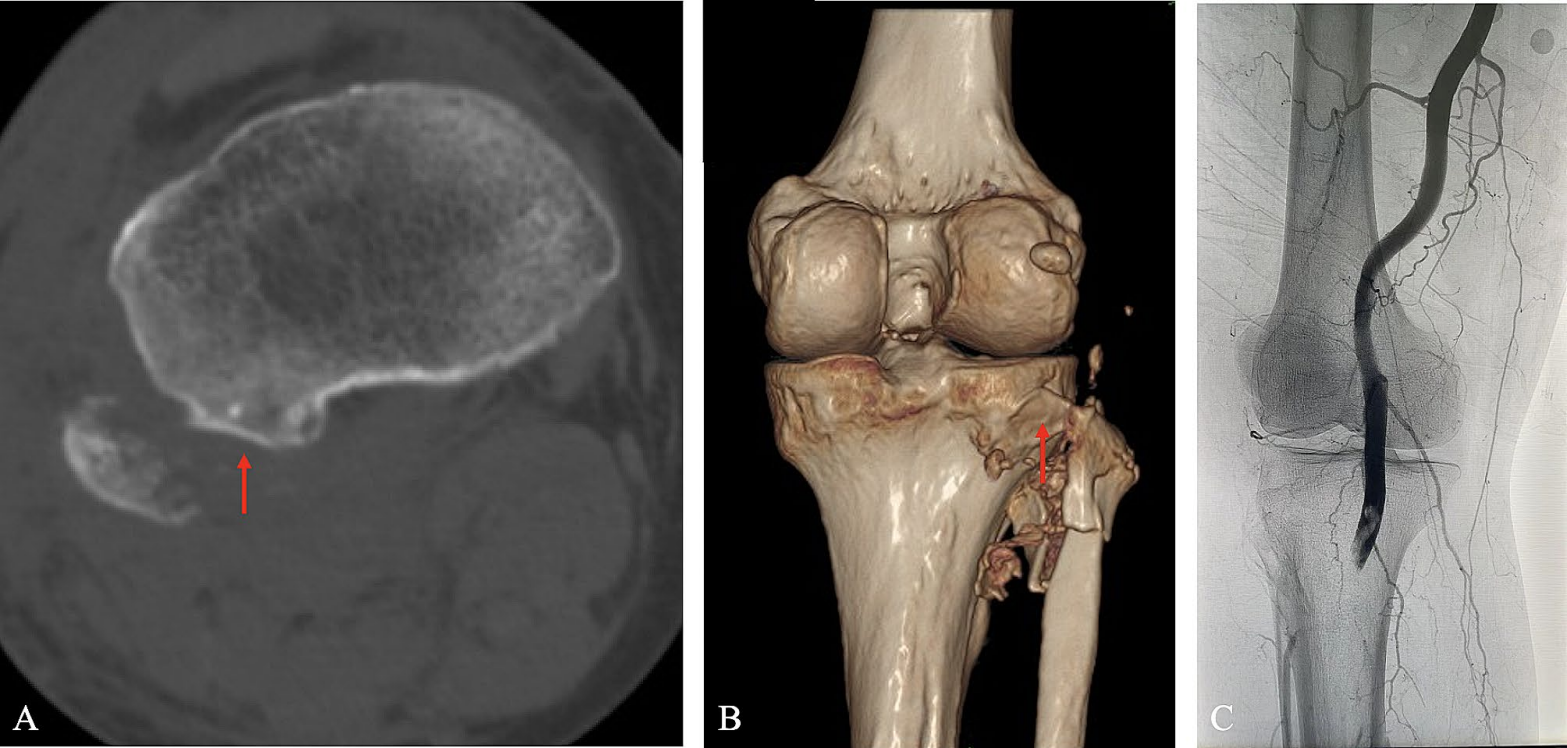
Traumatic proximal tibiofibular fracture and dislocation (anterolateral dislocation). Axial CT (**A**) and 3D reconstruction (**B**) imaging shows anterolateral dislocation of fractured fibular head (red arrow indicates the tibial articular surface of proximal tibiofibular joint). Angiography suggests popliteal artery injury (**C**)

**Fig. 2:**
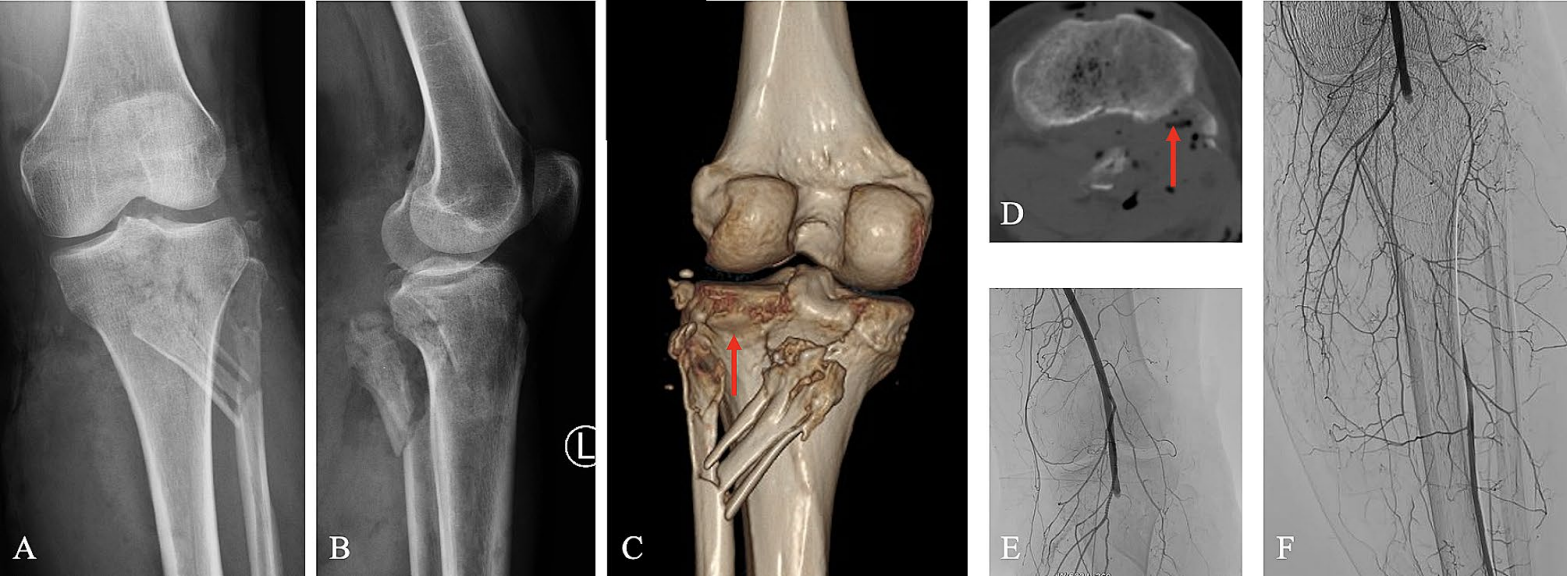
Traumatic proximal tibiofibular fracture and dislocation (posteromedial dislocation). AP view (**A**), lateral view (**B**), Axial CT (**D**) and 3D reconstruction (**C**) shows proximal fibula fracture with posteromedial dislocation of fibular head (red arrow indicates the tibial articular surface of proximal tibiofibular joint). Angiography (**E**, **F**) indicates popliteal artery injury

**Fig. 3:**
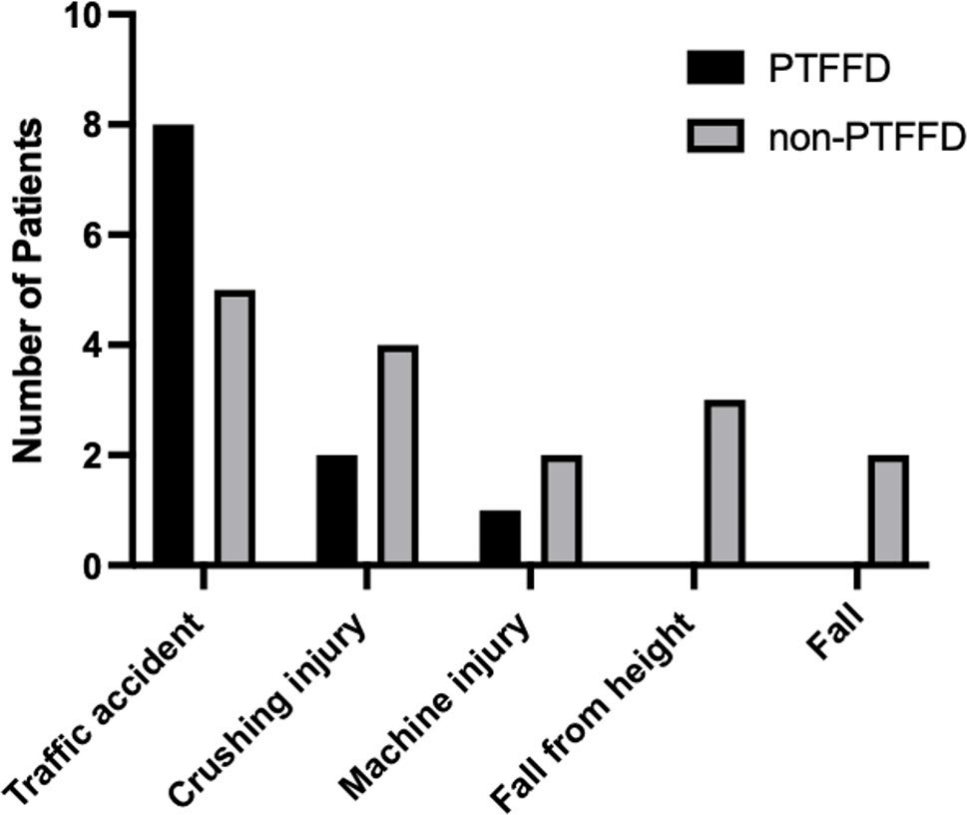
Mechanism of injury in both groups

Incidence of PTFD was 39.3% (11/28) in traumatic knee with vascular injuries, including 8 anterolateral dislocations (Figs. 1) and 3 posteromedial dislocations (Fig. 2). Three patients (3/11, 27.3%) in PTFD group had combined head, chest, or abdominal trauma, and 1 (1/16, 6.3%) in non-PTFD group. PTFD group had significantly more limbs with open injuries compared with non-PTFD group (10/11 VS 7/17, *p*<0.05).

In PTFD group, the main injury mechanism was traffic accident (8/11, 72.7%), followed by crush injury (2, 18.2%) and machine injury (1, 9.1%). In non-PTFD group, traffic accidents (5, 31.3%) and crush injuries (4, 25%) were the most common causes of injuries, followed by fall from height (3, 18.8%), machine injury (2, 12.5%) and fall (2,12.5%) (Fig. 3). Eight limbs in PTFD group and 15 limbs in non-PTFD group were diagnosed with popliteal artery injury by angiography, and the rest were partial branch injury below popliteal artery.

One patient in PTFD group died during hospitalization. The overall amputation rate among surviving patients was 29.6% (8/27 limbs), including 3 emergency amputations (2 in PTFD group and 1 in non-PTFD group) and 5 amputations after failed limb salvage (2 in PTFD group and 3 in non-PTFD group). Amputation rate of PTFD group was 40% (4/10), compared to 23.5% (4/17) in non-PTFD group. The difference between two groups was not statistically significant (*p*>0.05) (Table 1).

## Discussion

Traumatic PTFD is relatively uncommon and sparsely reported in literature, except for case reports [1, 3, 10, 14, 17–19]. Due to insufficient awareness, PTFD is easily overlooked or missed [3, 18]. A retrospective study conducted by Herzog et al. showed that incidence of proximal tibiofibular dislocation in tibial plateau fractures and tibial shaft fractures was only 1–2% [15]. However, PTFD is usually caused by high-energy trauma and is considered a marker of severe limb trauma. The incidence of PTFD in higher energy trauma was still unknown. Knee trauma combined with vascular injuries usually resulted from high-energy damage. Our investigation found the incidence of PTFD in traumatized knees with vascular injury was as high as 39.3% (11/28), which indicated that in severe knee trauma, the occurrence of PTFD might be a sign of vascular injury. All the 11 PTFDs occurred together with proximal tibial fractures or distal femoral fractures, making them easily overlooked or missed. In this study, none of the 11 cases had a clear diagnosis of PTFD in their records.

Eight limbs in PTFD group and 15 limbs in non-PTFD group were diagnosed with popliteal artery injury by angiography in this study. Popliteal artery is tethered proximally by adductor hiatus and distally by soleus arch in the knee joint, making it susceptible to damage during fractures and dislocations of the proximal tibiofibular joint.

PTFD group had significantly more limbs with open injuries compared with non-PTFD group (10/11 VS 7/17, *p*<0.05), suggesting that it might be caused by higher energy trauma. Similarly, we found that traffic accidents accounted for a higher proportion (8/11, 72.7%) in the PTFD group (Fig. 3).

According to the Ogden (1974) classification system [4, 7], traumatic PTFD can be divided into four categories: subluxation, anterolateral dislocation, posteromedial dislocation, and superior dislocation of the fibula head. Anterolateral dislocation was reported as the most common type. This was consistent with our results that 11 PTFDs included 8 anterolateral dislocations (Figs. 1) and 3 posteromedial dislocations (Fig. 2).

As a marker of severe limb trauma, the occurrence of PTFD may indicate the patient at higher risk of amputation [16]. However, reported amputation rate varies greatly in different studies [15, 16, 20]. In a retrospective study of 30 patients with proximal tibiofibular dislocation by Herzog et al. [15], only 2 patients underwent amputation due to a nonreconstructable extremity. The incidence of vascular injury was only 6.7% in their study [15], which might suggest a relatively lower trauma energy. In contrast, another retrospective analysis of 17 cases with proximal tibiofibular dislocations by Rajan et al. [16] reported that the incidence of vascular injury requiring surgical intervention was 29.4%, and the amputation rate was 47%. Gabrion et al [20] even reported a higher amputation rate of 62.5% in eight cases. In this present study, only one patient in PTFD group died. The overall amputation rate among surviving patients was 29.6% (8/27 limbs). Although there was no significant difference compared with non-PTFD group (23.5%), the PTFD group had a relatively high amputation rate of 40% (*p*>0.05) (Table 1). Our research focused on patients with high-energy knee trauma and vascular injury. This might explain why amputation rates were higher in both groups.

## Conclusions

PTFD was easily overlooked or missed. In traumatized knees with vascular injury, the incidence of PTFD was as high as 39.3%. The presence of PTFD might indicate severe knee trauma and the possibility of open injury. Although there was no significant difference compared with non-PTFD group, PTFD group had a relatively high amputation rate of 40%. Large sample size, prospective study will help further analysis to clarify the incidence of PTFD and its impact in high-energy knee trauma.

## Data Availability

Datasets used in this study are available from corresponding author (Bo Li) upon reasonable request.

## Abbreviations

PTFD: Proximal tibiofibular fracture and dislocation
CT: Computerized tomography
